# Supplementary material for: Metal-coated microsphere monolayers as surface plasmon resonance sensors operating in both transmission and reflection modes
Source: Sci Rep. 2019 Mar 6;9:3683. doi: 10.1038/s41598-019-40261-x (PMC6403218; doi:10.1038/s41598-019-40261-x)
Supplement: Supplementary file 1 — Additional simulation results [file 41598_2019_40261_MOESM1_ESM.docx]

**Supplementary Information**

**Metal-coated microsphere monolayers as surface plasmon resonance sensors operating in both** **transmission and reflection modes**

Cosmin Farcau^1,2^

*^1^National Institute for Research and Development of Isotopic and Molecular Technologies, 67-103 Donat Str., 400293 Cluj-Napoca, Romania*

*Email:cfarcau@itim-cj.ro*

*^2^Institute for Interdisciplinary Research in Bio-Nano-Sciences, Babes-Bolyai University, 42 T. Laurian, 400271, Cluj-Napoca, Romania*

**1. Role of triangular nanoparticles in the optical response of Ag-coated microsphere monolayers.**

Fig. S1 presents results of simulations of Ag-coated microsphere monolayers from which the Ag nanoparticles on the substrate were removed (red curve), compared to the whole structure (black curve). Triangular particles of two different sizes were considered: ones with 35 nm sides and others with 55 nm sides. Only minor differences are observed, confirming that the overall optical response is dominated by the sphere array and metal coating.


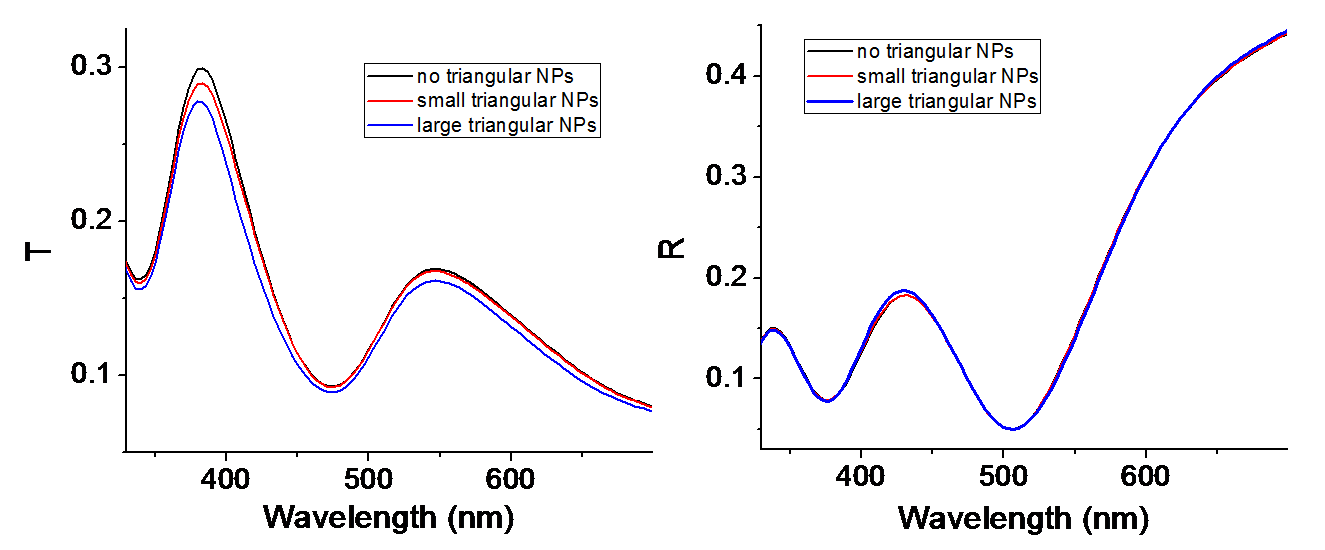


**Fig. S1.** Transmittance (left) and Reflectance (right) spectra of Ag-coated microspheres, for the structures without triangular nanoparticles (black curves) and whole structures with triangular particles of increasing size (red and blue curves).

**2. FOM_layer_ for gold-coated microsphere monolayers.**

Figure S2 presents FOM_layer_ for n=1.3 and t=5.0 nm, in the case of gold-coated microsphere monolayers. The structure is constructued as the one made with silver, discussed in the manuscript: 210 nm polystyrene spheres are coated by 45 nm of gold. Like for the silver structure, it can perform as optical sensor in both transmission and reflection modes.


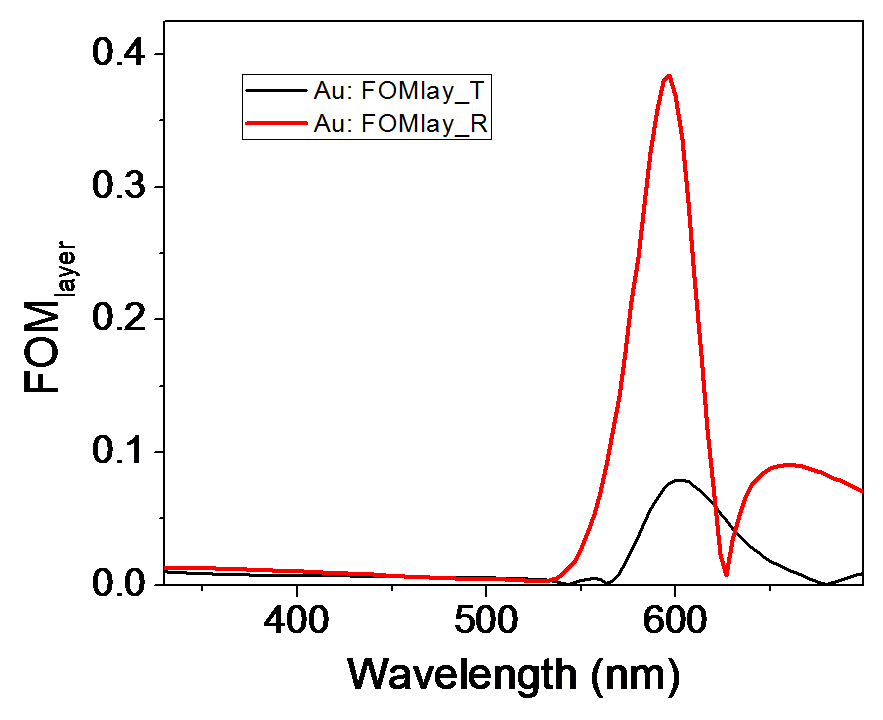


**Fig. S2.** *FOM_layer_* for gold-coated microspheres, in transmission and reflection modes. Adsorbed layer has a refractive index *n* = 1.3 and thickness *t* = 5 nm.
